# Susceptibility to *Mycobacterium ulcerans* Disease (Buruli ulcer) Is Associated with *IFNG* and *iNOS* Gene Polymorphisms

**DOI:** 10.3389/fmicb.2017.01903

**Published:** 2017-10-04

**Authors:** Stéphanie Bibert, Martin W. Bratschi, Samuel Y. Aboagye, Emilie Collinet, Nicole Scherr, Dorothy Yeboah-Manu, Christian Beuret, Gerd Pluschke, Pierre-Yves Bochud

**Affiliations:** ^1^Infectious Diseases Service, Department of Medicine, Lausanne University Hospital, University of Lausanne, Lausanne, Switzerland; ^2^Department of Medical Parasitology and Infection Biology, Swiss Tropical and Public Health Institute, Basel, Switzerland; ^3^University of Basel, Basel, Switzerland; ^4^Noguchi Memorial Institute for Medical Research, University of Ghana, Accra, Ghana; ^5^Spiez Laboratory, Federal Office for Civil Protection, Spiez, Switzerland

**Keywords:** immunogenetics, Buruli ulcer, *Mycobacterium ulcerans*, infectious diseases, single nucleotide polymorphism

## Abstract

Buruli ulcer (BU) is a chronic necrotizing disease of the skin and subcutaneous fat tissue. The causative agent, *Mycobacterium ulcerans*, produces mycolactone, a macrolide toxin, which causes apoptosis of mammalian cells. Only a small proportion of individuals exposed to *M. ulcerans* develop clinical disease, as surrounding macrophages may control the infection by bacterial killing at an early stage, while mycolactone concentration is still low. Otherwise, bacterial multiplication leads to in higher concentrations of mycolactone, with formation of necrotizing lesions that are no more accessible to immune cells. By typing a cohort of 96 Ghanaian BU patients and 384 endemic controls without BU, we show an association between BU and single nucleotide polymorphisms (SNPs) in *iNOS* (rs9282799) and *IFNG* (rs2069705). Both polymorphisms influence promoter activity *in vitro*. A previously reported SNP in *SLC11A1* (*NRAMP*, rs17235409) tended to be associated with BU. Altogether, these data reflect the importance of IFNG signaling in early defense against *M. ulcerans* infection.

## Introduction

Buruli ulcer (BU) is a necrotizing skin disease caused by *Mycobacterium ulcerans*, a slow growing mycobacterium, which can result in permanent functional disabilities (Junghanss et al., 2014). BU affects populations living in contact with stagnant or slow flowing water bodies in rural tropical regions from Africa, Asia, South America, and Australia. In endemic areas of West African countries (such as Côte d’Ivoire, Cameroon, Ghana, and Benin), the disease may be more prevalent than tuberculosis (TB) and leprosy with regionally up to 22% of the population infected (Amofah et al., 1993), mainly children between the age of 5 and 15 (Bratschi et al., 2013).

There is a wide range of clinical responses to *M. ulcerans* infection (Junghanss et al., 2014). Some individuals seem to be exposed without developing a clinically relevant infection (Diaz et al., 2006; Yeboah-Manu et al., 2012; Roltgen et al., 2014). Other individuals can develop clinical disease with spontaneous resolution (Gordon et al., 2011), while the remaining BU patients develop a chronic disease. Most lesions are located at the limbs; clinical presentations range from non-ulcerative (papules, nodules, plaques, or edema) to ulcerative forms (van der Werf et al., 1999). The WHO has introduced an additional classification, based on lesion size (World Health Organisation, 2012) [Category I: a single lesion < 5 cm in diameter; Category II: a single lesion measuring 5–15 cm in diameter; Category III: a single lesion > 15 cm in diameter, multiple lesions, lesion(s) at a critical site and osteomyelitis].

The extensive tissue destruction typically found in BU mainly results from the action of mycolactone, a cytotoxic and immunosuppressive macrolide toxin produced by *M. ulcerans* (George et al., 1999, 2000). Mycolactone increases expression of the pro-apoptotic regulator Bim in mammalian cells, driving them into apoptosis (Bieri et al., 2017). At low concentrations, mycolactone counteracts many functions of tissue-resident macrophages and monocytes by inhibiting the production of several cytokines and chemokines including TNF and IFNG (Simmonds et al., 2009; Torrado et al., 2010; Fraga et al., 2011). In addition, mycolactone suppresses dendritic cell (DC) maturation and reduces their ability to respond to stimulation, thus secondarily affecting T-cell activation (Pahlevan et al., 1999; Coutanceau et al., 2007; Boulkroun et al., 2010). Despite these immunosuppressive activities of mycolactone there is evidence that many exposed individuals do not develop clinical disease (Diaz et al., 2006; Yeboah-Manu et al., 2012; Roltgen et al., 2014). While in established lesions extracellular clusters of *M. ulcerans* are found in completely necrotic subcutaneous tissue (Ruf et al., 2016), an intra-macrophage growth phase may play an important role in the early phase of the infection (Torrado et al., 2007). The necrotic core of early BU lesions is surrounded by a belt of infiltrating leukocytes consisting mainly of macrophages and T-cells (Bolz et al., 2016), which appear to be activated (Peduzzi et al., 2007). Analyses with peripheral blood mononuclear cells (PBMCs) stimulated with mycobacterial antigens have indicated that susceptibility to BU may reflect individual differences in the nature of the cellular immune response. While BU patients showed a T-helper-2 type response, unaffected household contacts predominately produced a T-helper-1 cytokine (IFNG and IL-2) pattern (Gooding et al., 2002). An adequate T-helper-1 cell mediated activation of macrophages at an early stage of the disease may thus lead to curing, as also suggested by the observation of an inverse correlation between the expression level of IFNG and the severity of BU lesions (Prevot et al., 2004). Furthermore, in a *M. ulcerans* mouse footpad infection model it was found that IFNG knockout mice display a faster disease progression compared to wild type mice (Bieri et al., 2016). This accelerated progression was reflected by faster and more extensive tissue necrosis, as well as by a significantly higher bacterial burden.

The critical balance between effective immune defense against *M. ulcerans* and the immunosuppressive effects of mycolactone may be influenced by host genetic factors. To date, only the rs17235409 single nucleotide polymorphism (SNP) of the natural resistance-associated macrophage protein gene *SLC11A1* (*NRAMP1*) (Stienstra et al., 2006; Barogui et al., 2016) and the rs1333955 SNP of the autophagy-related *PARK2* gene (Capela et al., 2016) have been associated with susceptibility to BU. Yet, studies have reported robust associations between a range of additional host polymorphisms and susceptibility to other mycobacteria such as *M. tuberculosis* (Bellamy, 1998, 1999, 2000; Goldfeld et al., 1998) and *M. leprae* (Lagrange and Abel, 1996; Abel et al., 1998). We hypothesized that some of these polymorphisms can also influence the course of infection due to *M. ulcerans*. Therefore, we conducted a case-control candidate gene study to identify polymorphisms affecting susceptibility to BU.

## Materials and Methods

### Ethics Statement

Ethical approval for the collection and testing of human blood samples was obtained from the institutional review board of the Noguchi Memorial Institute for Medical Research (Federal-wide Assurance number FWA00001824). Written informed consent was obtained from all individuals involved in the study. Parents or guardians were informed of the risks of participating their children in the study and provided written consent on behalf of them. PBMCs were prepared from healthy Caucasian volunteers who provided written informed consent for genetic and immunological studies (protocol “Etudes fonctionnelles,” Lausanne Ethics Committee #130/08 and protocol “Vaxigen B,” #30/08) and were genotyped for rs2069705.

### Study Patients

Patients were included from villages within the Obom subdistrict of the Ga-South district of Ghana. This sub-district is one of the major BU endemic communities along the Densu River Basin. Study participants (**Table 1**) included 96 laboratory (*IS2404* PCR) confirmed BU patients (57 females and 39 males) as well as four age-, sex-, ethnicity and home village-matched controls for each patient (384 control individuals). None of the control participants had a history of mycobacterial infection. All the study population was HIV-negative. Demographic data as well as history of known previous mycobacterial infections were recorded for all participants. Lesions were classified following the WHO classification (World Health Organisation, 2012).

**Table 1:** Baseline characteristics of BU patients.

	BU patients (*N* = 96)	Controls (*N* = 384)
**Sex**		
Male	39 (0.41)	156 (0.41)
Female	57 (0.59)	228 (0.59)
Age^1^ median (IQR)	13 (26)	13 (23)
**Clinical form^2^**		
Ulcer	82 (0.85)	
Nodule	9 (0.09)	
Plaque	5 (0.04)	
Edema	6 (0.06)	
Osteomyelitis	2 (0.02)	
**Location of lesion**		
Lower limbs	67 (0.70)	
Upper limbs	19 (0.20)	
Head, trunk, buttocks	10 (0.10)	
**Category of lesion^3^**		
(I) Single lesion < 5 cm in diameter	42 (0.44)	
(II) Single lesion 5–15 cm in diameter	11 (0.11)	
(III) Single/multiple lesions > 15 cm in diameter	43 (0.45)	
**Number of lesions**		
Single	91 (0.95)	
Multiple	5 (0.05)	


*Data are number of study participants unless otherwise indicated.*

*^1^At cohort registration.*

*^2^Eight patients had more than one clinical presentation (ulcer and edema [*N* = 5], ulcer and plaque [*N* = 1], ulcer and osteomyelitis [*N* = 2]).*

*^3^The WHO definition of categories is provided in the introduction.*

### DNA Extraction, SNP Selection, and Genotyping

Whole blood samples were collected into 5 ml EDTA vacutainer tubes, transported under cold condition to the Noguchi Memorial Institute for Medical Research and stored at -80°C until further analysis.

DNA was extracted from 450 μL of human blood using the MagNA Pure 96 Instrument (Roche) according to manufacturer instructions.

A total of nine SNPs from six genes were selected on the following criteria, (1) SNPs previously associated with susceptibility to BU (2) minor allele frequency (MAF) > 0.05 in the YRI population (Yoruba in Ibadan, Nigeria) (3) SNPs predicted or known to influence the gene expression or the protein function. These SNPs were genotyped from a customized GoldenGate SNP Genotyping Assay (Illumina, San Diego, CA, United States). Genotypes were assigned on a BeadXpress Reader according to standard protocols and quality controls. SNPs that presented low quality cluster separation scores (cutoff of 0.20) and call rate (<95.0%) or any deviations from Hardy–Weinberg equilibrium were discarded. The most frequent allele was used as the reference allele.

The IFNG microsatellite rs3138557 was determined by capillary electrophoresis after amplification using forward (CACGACGTTGTAAAACGACGCTGTCATAATAATATTCAGA) and reverse (CGAGCTTTAAAAGATAGTTCC) primers.

### Statistical Analysis

Statistical analyses were performed using Stata (version 14.1, StataCorp LP, College Station, TX, United States). Linkage disequilibrium (LD) and Hardy–Weinberg equilibrium test were assessed using the programs pwld and genhw, respectively, both implemented in Stata. The association of polymorphisms with BU susceptibility or lesion severity was performed by logistic regression, assuming an additive model of inheritance.

### Bacterial Lysates

The *M. ulcerans* strain S1013 has been isolated in 2010 from the ulcerative lesion of a Cameroonian BU patient (Bratschi et al., 2013). The *M. marinum* strain S1245 corresponds to the ATCC^®^ BAA535^TM^ strain M. Bacteria were cultivated for several weeks in BacT/ALERT^®^ MB medium complemented with enrichment fluid (BioMérieux), washed several times, heat-inactivated at 95°C and sonicated in 0.9% NaCl. Debris and non-lysed cells were removed by centrifugation 5 min at 10.000 × *g* and 4°C, and the supernatant used for stimulation experiments.

### Peripheral Blood Mononuclear Cell Isolation and RT-PCR Analysis

Peripheral blood mononuclear cells were prepared from healthy Caucasian volunteers who provided written informed consent for genetic and immunological studies and were genotyped for *rs2069705*. Briefly, whole blood diluted in PBS was overlaid above Ficoll-Paque Plus (GE Healthcare, Uppsala, Sweden) and mononuclear cells extracted by gradient density centrifugation. Viability, as determined by trypan blue exclusion, was >90%. PBMCs were stimulated with lipopolysaccharide (LPS) 100 ng/ml or killed mycobacterial lysates (5 μg) for various durations. Total RNA was isolated from PBMCs using an RNeasy Mini kit and the automated QIAcube (Qiagen, Hombrechtikon, Switzerland). Quality and quantity of the RNA was measured using the Xpose reader (Trinean, Belgium). Total RNA (150–250 ng) was reverse transcribed using the QuantiTect Reverse Transcription kit (Qiagen). The relative levels of IFNG, and IL-10 transcripts were determined by RT-PCR, with a 7500 Fast real-time PCR system (Applied Biosystems), using the Power SYBR green PCR master mix (Applied Biosystems) with the following primers designed using the Primer 3 software and validated by BLAST analysis: IFNG Forward primer 5′-GAGTGTGGAGACCATCAAGGAAG-3′ and reverse primer 5′-TGCTTTGCGTTGGACATTCAAGTC-3′, IL-10 Forward primer 5′-AACAAGAGCAAGGCCGTGG-3′ and reverse primer 5′-GAAGATGTCAAACTCACTCATGGC-3′. HPRT expression was not influenced by cell stimulation and was used as a housekeeping gene with the following primers HPRT Forward primer 5′-GAACGTCTTGCTCGAGATGTG-3′ and reverse primer 5′-CCAGCAGGTCAGCAAAGAATT-3′. The relative levels of mRNA expression to HPRT were determined by the 2^(-ΔΔ*C*_t_)^ method and expressed in arbitrary units (A.U.).

### Enzyme-Linked Immunosorbent Assay

IFNG produced in the supernatant of stimulated PBMCs were assayed by enzyme-linked immunosorbent assay (ELISA) (Affymetrix eBioscience). According to the manufacturer’s recommendations, capture antibody was coated onto 96-well plate (Nunc Maxisorp) overnight at 4°C. After washing with buffer containing PBS, 0.05% Tween 20, wells were blocked with 1x ELISA/ELISPOT diluents for 1 h at room temperature. Diluted samples and standard were added overnight at 4°C. After washing, detection antibody was applied for 1 h at room temperature, followed by five washes and incubation with diluted horseradish peroxidase-conjugated avidin for 30 min at room temperature. After washing, plates were treated with tetramethyl benzidine substrate and the enzymatic reaction was stopped by H_2_SO_4_ after 15 min. Plates were finally read at 450 nm with a reference wavelength of 570 nm. Quantification of IFNG concentration in the supernatant was then normalized by using the total amount of RNA extracted from PBMCs. Each experiment was performed in duplicate, and the mean of each replicate was used in expressing final results as mean ± standard error.

### Construction of Luciferase Reporter of the iNOS Promoter

A 1464 base pair fragment from -1385 to +79 base pair of the human iNOS promoter was amplified by PCR, from genomic Caucasian DNA with wild type G *rs9282799* allele. PCR amplification with Phusion enzyme (Thermoscientific) was performed using 30 cycles with denaturation, annealing and extension conditions, respectively at 98°C for 10 s, 60°C for 10 s and 72°C for 1 min using a sense primer tailed with a MluI site (5′-GCACGCGTCTAAGCCGCAGCATTGAGCC-3′) and an antisense primer tailed with a XhoI restriction site (5′-GCCTCGAGCCCAGTCCCCTCATCAAAGG-3′). Restriction enzyme sites are underlined. PCR products were then cloned into the MluI/XhoI-cut pGL3 basic vector (Promega) to generate the wild type iNOS-firefly luciferase construct. The mutant *rs9282799* A allele was introduced into the wild type iNOS-firefly luciferase construct by PCR-based method using the Quickchange II site directed mutagenesis (Agilent technologies) and mutagenic primers (5′-ACCCTTGATCTCACCATCCCAACACTTTGCTACC-3′, 5′-GGTAGCAAAGTGTTGGGATGGTGAGATCAAGGGT-3′. All constructs were sequenced.

### Cell Culture, Transient Transfection, and Luciferase Gene Reporter Assay

HeLa cells (ATCC^®^ -CCL-2) at 70–80% confluence, cultured in DMEM (Gibco Laboratories) supplemented with 10% fetal bovine serum, 100 U/ml penicillin, and 100 mg/ml streptomycin at 37°C in a humidified atmosphere containing 5% CO_2_ in 24-well plates were transfected with 500 ng of wild type or mutant iNOS-firefly luciferase construct and 2 μl of JetPEI together with 5 ng of a renilla luciferase construct that was used to normalize transfection efficiencies. A mix of PMA 50 nM/ionomycin 0.5 ug/ml, were added at different time prior to luciferase assay. Cells were harvested, washed with PBS and lysed. After centrifugation, 50 μl of supernatant was collected and luciferase activity was measured by a Synergy H1 luminometer (Biotek) using a dual luciferase reporter assay system (Promega) according to the protocol supplied by the manufacturer. After correction for transfection efficiency (Renilla activity), luciferase activity was expressed as relative light units and compared with the positive control (pGL3). Each experiment was performed in duplicate on three separate occasions, and the mean of each replicate was used in expressing final results as mean ± standard error.

## Results

The study included 96 Ghanaian BU patients with *IS2404* PCR-proven *M. ulcerans* infection and 384 controls without BU, matched for age, sex, residence, and ethnicity (**Table 1**). The median age at BU diagnosis was 13 years [interquartile range (IQR) 26]; 41% were males and 59% females. Most of the BU patients had a single lesion and the most frequent clinical presentation was ulcerative (82%). Limbs were mainly affected (90%) and WHO categories I (44%) and III (43%) accounted for the majority of lesions.

Among the nine SNPs tested, three were or tended to be more frequent among BU cases than controls (**Table 2** and Supplementary Table S1). Carriage of the A allele at *iNOS* rs9282799 (OR = 1.99, 95% CI 1.22–3.26, *p* = 0.006, additive mode of inheritance) and of the G allele at *IFNG* rs2069705 (OR = 1.56, 95% CI 1.14–1.99, *p* = 0.007) was associated with BU. In addition, carriage of the A allele at *SLC11A1* (*NRAMP1*) rs17235409 tended to be associated with BU (OR = 1.63, 95% CI 0.99–2.70, *p* = 0.06).

**Table 2:** Independent genetic factors associated with BU susceptibility among candidate genes.

Gene/SNP	Genotype	All^∗^	Controls	BU patients	OR (95% CI)	*p*^1^
*iNOS*	GG	400 (0.85)	329 (0.88)	71 (0.76)		
rs9282799	GA	64 (0.14)	43 (0.11)	21 (0.22)		
	AA	6 (0.01)	4 (0.01)	2 (0.02)	1.99 (1.22–3.26)	**0.006**
*iNOS*	GG	316 (0.67)	246 (0.65)	70 (0.74)		
rs8078340	GA	139 (0.29)	118 (0.31)	21 (0.22)		
	AA	20 (0.04)	16 (0.04)	4 (0.04)	0.74 (0.48–1.14)	0.17
*IFNG*	AA	131 (0.28)	113 (0.30)	18 (0.19)		
rs2069705	AG	239 (0.50)	191 (0.50)	48 (0.50)		
	GG	103 (0.22)	74 (0.20)	29 (0.31)	1.56 (1.14–2.17)	**0.007**
*IFNG*		237 (0.50)	180 (0.47)	57 (0.60)		
rs3138557		189 (0.40)	155 (0.41)	34 (0.36)		
(CA)_14/15_		51 (0.10)	47 (0.12)	4 (0.04)	0.60 (0.42–0.87)	**0.007**
*SLC11A1*	GG	394 (0.83)	323 (0.85)	71 (0.75)		
rs17235409	GA	78 (0.16)	54 (0.14)	24 (0.25)		
	AA	4 (0.01)	4 (0.01)	0 (0)	1.63 (0.99–2.70)	0.06
*PARK2*	AA	117 (0.25)	96 (0.26)	21 (0.23)		
rs1040079	AG	249 (0.53)	191 (0.51)	58 (0.63)		
	GG	101 (0.22)	88 (0.23)	13 (0.14)	0.87 (0.62–1.21)	0.41
*VDR*	AA	290 (0.61)	229 (0.60)	61 (0.64)		
rs731236	AG	156 (0.33)	126 (0.33)	30 (0.32)		
	GG	29 (0.06)	25 (0.07)	4 (0.04)	0.84 (0.57–1.23)	0.37
*VDR*	AA	229 (0.53)	191 (0.55)	38 (0.44)		
rs7975232	AC	161 (0.37)	122 (0.35)	39 (0.45)		
	CC	42 (0.10)	33 (0.10)	9 (0.11)	1.30 (0.92–1.84)	0.13
*NOD2*	GG	184 (0.39)	148 (0.39)	36 (0.38)		
rs9302752	GA	217 (0.46)	174 (0.46)	43 (0.45)		
	AA	72 (0.15)	56 (0.15)	16 (0.17)	1.07 (0.78–1.48)	0.68
*NOD2*	GG	252 (0.53)	196 (0.51)	56 (0.59)		
rs7194886	GA	183 (0.39)	154 (0.41)	29 (0.31)		
	AA	39 (0.08)	29 (0.08)	10 (0.10)	0.90 (0.63–1.28)	0.55


*OR stands for odds ratio, CI for confidence interval. All for total number of included individuals comprising BU patients and controls. The significant *p*-value after Bonferroni correction (considering nine different tests) was 0.0056. ^∗^The numbers of genotypes that passed quality control may be smaller than the total number of patients tested. Bold values indicate a difference with *p* < 0.05.*

Since the *IFNG* rs2069705 is known to be in LD with the variable nucleotide tandem repeat (VNTR) rs3138557 (variable length CA repeats located in the first intron of the *IFNG* gene), we genotyped in a second step the *rs3138557* VNTR to determine whether a specific allelic length was associated with BU at a higher level of significance than rs2069705. Taken separately, no allelic length showed a more significant association than rs2069705 (Supplementary Table S2). However, the shorter allelic lengths (CA)_14_ and (CA)_15_ showed an association with BU (OR = 0.60, CI 0.42–0.87, *p* = 0.007, **Table 2**), when considered together.

Since the polymorphisms associated with BU were located within the promoter regions of *IFNG* and *iNOS*, we hypothesized that they may influence the expression of these genes, and the subsequent activation of immune defense mechanisms against *M. ulcerans*. To verify this, we analyzed the expression of IFNG mRNA in fresh PBMCs from healthy Caucasian volunteers carrying the different rs2069705 alleles. PBMCs were stimulated with either LPS or lysates of *M. ulcerans* or *M. marinum* cells, assuming that the effect of this SNP is similar in African and Caucasian ethnicities.

**FIGURE 1:**
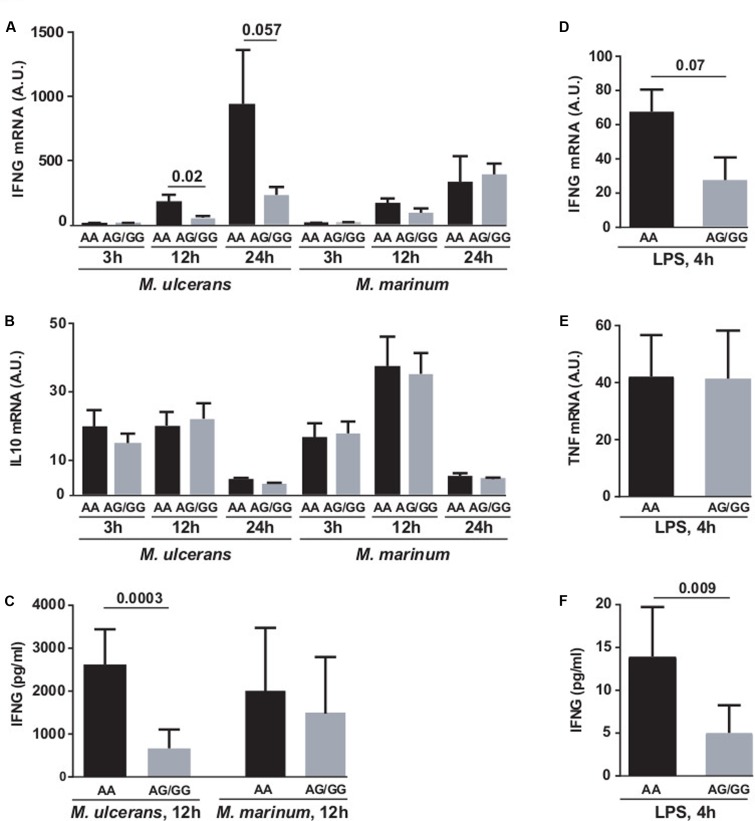
Functional impact of *IFNG* rs2069705 polymorphism on IFNG expression. PBMCs from healthy Caucasian individuals were stimulated with *M. ulcerans* or *M. marinum* lysate **(A–C)** or LPS **(D–F)** for the indicated periods of time. The *IFNG* mRNA and protein expression was measured by RT-qPCR **(A,D)** and ELISA **(C,F)**. As controls, the IL10 or TNF mRNA expression were determined by RT-qPCR **(B,E)**. Data are grouped according to *IFNG* rs2069705 A alleles [2 copies of the A allele, black (*n* = 5) and 1 copy of the A allele (*n* = 6) or 2 (*n* = 2) copies of the G allele (gray)]. A.U., arbitrary units.

The production of IFNG was not changed by stimulation with *M. marinum*. However, carriage of the G allele of rs2069705 was associated with reduced production of IFNG mRNA (**Figure 1A**), after 3, 12, and 24 h incubation with the *M. ulcerans* strain S1013 lysate, with an additive effect (GG < GA < AA). The production of IFNG as measured by ELISA was also reduced in the presence of the G allele at rs2069705 (**Figure 1C**). In contrast, no effect was seen for IL-10 mRNA (**Figure 1B**). Also in LPS stimulated PBMCs carriage of the G allele was associated with a decreased production of both IFNG mRNA (**Figure 1D**) and IFNG (**Figure 1F**). As a control, the production of TNF mRNA was not significantly influenced by the rs2069705 genotype (**Figure 1E**).

**FIGURE 2:**
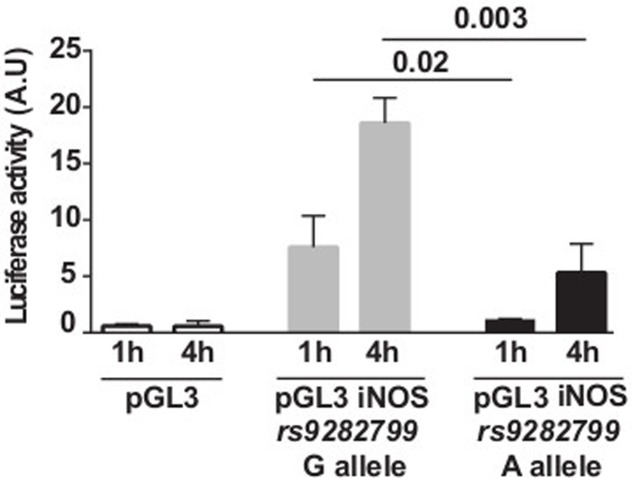
Functional impact of iNOS rs9282799 polymorphism on promoter activity. Transcriptional activation of the *iNOS* promoter in response to PMA/ionomycin monitored by normalized luciferase activity of the empty (white), G containing (gray), and A containing (black) constructs transfected into the HeLa cell line. A.U., arbitrary units.

While the *IFNG* rs2069705 polymorphism has been detected in both Caucasians and Africans, the *iNOS* rs9282799 A allele seems to be restricted to African populations (Yates et al., 2016). Therefore, we used a gene reporter assay to evaluate the influence of *rs9282799* on *iNOS* promoter activity. The rs9282799 G to A substitution was introduced by site directed mutagenesis into a Caucasian wild type *iNOS* cDNA and the 5′ flanking region of the gene was fused upstream of a luciferase reporter gene. In a transient luciferase reporter gene assay, HeLa cells transfected with the rs9282799 G containing fragment of the iNOS promoter showed a higher induction of luciferase activity in response to PMA/ionomycin as compared with the empty vector and the rs9282799 A containing construct (**Figure 2**).

## Discussion

In this case control association study, we tested the involvement of nine SNPs within six candidate genes on the incidence of BU. Two SNPs, one in *IFNG* and one in *iNOS*, were associated with BU. Another SNP in *NRAMP1* showed a trend toward an association.

The predominant paradigm for many years has been that the production of IFNG by helper T-cells is a key driver in the host defense against mycobacterial infections (Flynn and Chan, 2001; Stienstra et al., 2001). IFNG activation increases the capacity of macrophages to kill intracellular mycobacteria by enhancing antimicrobial effector pathways, including iNOS, phagosomal maturation and autophagy, all of which play critical roles in the clearance of mycobacteria (Nunes-Alves et al., 2014). This evidence initially derived from studies in IFNG knockout mice, which were shown to be more susceptible to TB than WT mice (Cooper et al., 1993; Flynn et al., 1993). In humans, resting macrophages are unable to kill intracellular pathogens such as *M. tuberculosis*, while IFNG-activated macrophages can (Zhang et al., 1995; Lin et al., 1996). Further evidence for the role of IFNG comes from the characterization of several types of primary immune deficiencies in which mutations in the IFNG receptor were shown to confer Mendelian susceptibility to mycobacterial disease, i.e., susceptibility to non-TB mycobacterial infections (Newport et al., 1996) or to disseminated BCG disease (Jouanguy et al., 1996). Similarly, several studies have highlighted a major role for IFNG in the immune response to *M. ulcerans*. IFNG knockout mice were found to be more susceptible than wild type mice in a footpad model of *M. ulcerans* infection (Bieri et al., 2016). In humans, studies analyzing the cytokine production of PBMCs after stimulation by *M. ulcerans* showed that BU patients produced different cytokine patterns as compared to healthy controls, mainly characterized by low levels of IFNG. Within the same line, suppression of IFNG responses has been observed in patients with active *M. ulcerans* infection and this suppression resolved after surgical excision of the BU lesions (Yeboah-Manu et al., 2006). In BU patients, reduced IFNG production may be due to the immunosuppressive effect of mycolactone. However, at an early stage of infection, the individual ability to produce IFNG may determine the outcome of the disease toward mycobacterial clearance versus chronic infection (Gooding et al., 2002; Prevot et al., 2004). Subtle differences in the ability to mount efficient IFNG responses and subsequent efficient anti-mycobacterial defense reactions at an early stage of *M. ulcerans* infection may result from genetic polymorphisms.

We report here for the first time an association between the G allele of the IFNG promoter SNP rs2069705 and susceptibility to BU. The same polymorphism has been associated with susceptibility to TB in a study of 625 West African cases and 589 controls (Cooke et al., 2006). These results are consistent with two smaller studies, one including 45 Italian patients with TB and 97 controls (Lio et al., 2002), and the other including 113 Spanish patients with TB and 207 controls (Lopez-Maderuelo et al., 2003), that both showed an association between the A allele of rs2430561 (a SNP which is in LD with rs2069705) and susceptibility to the disease.

The association between IFNG rs2069705 and mycobacterial disease is further supported by functional evidence. We demonstrate that the G allele of rs2069705 is associated with reduced IFNG expression levels. This observation is also consistent with previous functional analyses showing that specific allelic lengths from an intronic polymorphic microsatellite, which are in LD with both rs2069705 and rs2430561, can modulate *in vitro* levels of IFNG production (Pravica et al., 1999, 2000). Yet, it is difficult to determine which polymorphism is the causative one, because most of these variants are co-inherited, and the number of individuals carrying discrepant genotypic patterns would be too low to ensure discriminant analyses.

Innate immune cells produce reactive oxygen and NO which promotes the destruction of intracellular pathogens (Nathan and Shiloh, 2000). NO production by macrophages requires iNOS expression which is induced by recognition of conserved pathogen-associated molecular patterns (PAMPs) and/or inflammatory cytokines, such as IFNG and IL-1β (Chartrain et al., 1994; Knowles and Moncada, 1994; Morris and Billiar, 1994; Nathan, 1997; Taylor et al., 1998; Weinberg, 1998; Fang, 2004). In humans, NO produced by human macrophages and epithelial cells seems to play a critical role in the clearance of mycobacteria (Rich et al., 1997) and its amount is increased in TB patients compared to healthy controls (Nicholson et al., 1996; Wang et al., 1998) due to an up-regulation of iNOS (Wang et al., 1998). Also the intracellular proliferation of *M. ulcerans* seems to be controlled via a NO dependent mechanism associated with maturation and acidification of phagosomes (Torrado et al., 2010).

Here, we identified for the first time an association between the A allele of the *iNOS* promoter SNP rs9282799 and susceptibility to BU. By using a luciferase gene reporter assay, we show that the A allele of rs9282799 is associated with a reduced iNOS promoter activity suggesting that it causes a decreased NO production. The same SNP has been previously associated with susceptibility to TB in a study of 395 cases and 405 controls in South Africa (Moller et al., 2009). Smaller studies reported associations with two microsatellites (CCTTT AND TAAA) and several other promoter SNPs within *iNOS* and susceptibility to TB (Jamieson et al., 2004; Gomez et al., 2007; Moller et al., 2009; Velez et al., 2009b), leprosy (Jamieson et al., 2004) and malaria (Burgner et al., 1998; Kun et al., 1998; Hobbs et al., 2002; Boutlis et al., 2003). Some of these studies also provided functional evidence of differential gene expression (Warpeha et al., 1999) or NO production (Hobbs et al., 2002; Burgner et al., 2003). Although these studies are together suggestive of a significant role of such variants with regard to infections, they have been performed in different populations and did not include many overlapping SNPs. Because the *iNOS* gene is highly polymorphic with major differences in allele frequencies between ethnicities, it is difficult to reconcile data and conclusively identify the causative polymorphism. Large cohort studies will be needed to solve these issues.

We also observed a trend toward an association between the *SLC11A1* (*NRAMP1*) rs17235409 polymorphism (D543N) and BU. NRAMP1 is exclusively expressed in the late endosomal and lysosomal membrane of macrophages and translocates to the phagosomal membranes where it transports divalent cations (Jabado et al., 2000). This induces microbicidal activities in infected macrophages. Our observation is consistent with a study of 182 West African cases and 191 controls in Ghana that previously reported an association between this rs17235409 polymorphism and BU (Stienstra et al., 2006). Four *NRAMP1* polymorphisms [a 4-base-pair *rs17235416* indel in the 3′ untranslated region, a 5′(GT)n microsatellite in the 5′ promoter region, an intron 4 rs3731865 SNP and an exon 15 rs17235409 SNP] have been previously associated with susceptibility or resistance to TB among Africans (Bellamy et al., 1998; Velez et al., 2009a). Furthermore, three of these polymorphisms (rs17235416, the microsatellite and rs3731865) were associated with clinical presentation of leprosy in west Africans (Meisner et al., 2001).

Finally, we failed to confirm the recently reported association between rs1040079 polymorphism in *PARK2* and susceptibility to BU (Capela et al., 2016).

Taken together, our study shows for the first time an association between polymorphisms in the *IFNG* and *iNOS* genes and susceptibility to BU. This association is sensible, since these SNPs are known to influence the innate immune response against mycobacteria by modulating the respective gene expression. As many other genetic association studies, our study is limited by a relative small sample size. While future functional studies are required to establish a clear link between the polymorphisms and effective mycobacterial killing, our results support the hypothesis that in the early stage of a *M. ulcerans* infection, when mycolactone levels are still low, the rate of multiplication of the mycobacteria in macrophages attracted to the infected tissue may be the most critical determinant for the outcome of the infection. If a critical mass of mycolactone producing *M. ulcerans* cells can develop, host cells are killed and infiltrating leukocytes can no longer reach the clusters of extracellular bacteria residing in the necrotic tissue (Ruf et al., 2017), resulting in the chronic necrotizing disease BU.

## Author Contributions

SB, MB, DY-M, GP, and P-YB designed the study. SB, EC, and NS performed the experiments and analyzed the data. P-YB and GP obtained funding, supervised the study and analyzed the data. MB, SA, NS, DY-M, and CB collected or processed clinical data and/or samples. SB, MB, GP, and P-YB wrote the manuscript with input from all co-authors. All authors critically revised the manuscript and approved its final version.

## Conflict of Interest Statement

The authors declare that the research was conducted in the absence of any commercial or financial relationships that could be construed as a potential conflict of interest.
