# Supplementary material for: Susceptibility to Mycobacterium ulcerans Disease (Buruli ulcer) Is Associated with IFNG and iNOS Gene Polymorphisms
Source: Front Microbiol. 2017 Oct 4;8:1903. doi: 10.3389/fmicb.2017.01903 (PMC5632961; doi:10.3389/fmicb.2017.01903)
Supplement: Supplementary file 1 [file Table_1.DOCX]

| **Gene/SNP** | **HWE** |  | **Alleles** | **MAF^1^** |  | **Cohort** | |
| --- | --- | --- | --- | --- | --- | --- | --- |
|  |  |  |  |  |  | **N** | **MAF** |
| IFNG | 0.76 |  | G | 0.44 |  | 445 | 0.47 |
| *rs2069705* |  |  | A | 0.56 |  | 501 | 0.53 |
| iNOS | 0.07 |  | G | 0.95 |  | 864 | 0.92 |
| *rs9282799* |  |  | A | 0.05 |  | 76 | 0.08 |
| iNOS | 0.35 |  | G | 0.78 |  | 771 | 0.81 |
| *rs8078340* |  |  | A | 0.22 |  | 179 | 0.19 |
| NRAMP1 | 0.95 |  | G | 0.93 |  | 866 | 0.91 |
| *rs17235409* |  |  | A | 0.07 |  | 86 | 0.09 |
| PARK2 | 0.14 |  | A | 0.55 |  | 483 | 0.52 |
| *rs1040079* |  |  | G | 0.45 |  | 451 | 0.48 |
| VDR | 0.20 |  | A | 0.73 |  | 736 | 0.77 |
| *rs731236* |  |  | G | 0.27 |  | 214 | 0.23 |
| VDR | 0.09 |  | A | 0.67 |  | 619 | 0.72 |
| *rs7975232* |  |  | C | 0.33 |  | 245 | 0.28 |
| NOD2 | 0.54 |  | G | 0.65 |  | 585 | 0.62 |
| *rs9302752* |  |  | A | 0.35 |  | 361 | 0.38 |
| NOD2 | 0.48 |  | G | 0.76 |  | 687 | 0.72 |
| *rs7194886* |  |  | A | 0.24 |  | 261 | 0.28 |

**S1 Table. Genotype distribution of SNPs in supercandidate genes.** SNP stands for single nucleotide polymorphism, MAF for minor allele frequency, HWE for Hardy-Weinberg equilibrium. **^1^**HapMap MAFs were calculated for YRI population.
