# Supplementary material for: Susceptibility to Mycobacterium ulcerans Disease (Buruli ulcer) Is Associated with IFNG and iNOS Gene Polymorphisms
Source: Front Microbiol. 2017 Oct 4;8:1903. doi: 10.3389/fmicb.2017.01903 (PMC5632961; doi:10.3389/fmicb.2017.01903)
Supplement: Supplementary file 2 [file Table_2.docx]

|  |  |  |  |  |  |  |  |  |  | |  |
| --- | --- | --- | --- | --- | --- | --- | --- | --- | --- | --- | --- |
| Gene/SNP |  | Genotype |  |  | All | Controls | BU patients |  | OR(95% CI) | P |  |
|  |  |  |  |  |  |  |  |  |  |  |  |
| *IFNG* |  |  |  |  | 390 (0.82) | 310 (0.81) | 80 (0.84) |  |  |  |  |
| rs3138557 |  |  |  |  | 82 (0.17) | 67 (0.18) | 15 (0.16) |  |  |  |  |
| (CA)_14_ |  |  |  |  | 5 (0.01) | 5 (0.01) | 0 |  | 0.77 (0.43-1.36) | 0.37 |  |
|  |  |  |  |  |  |  |  |  |  |  |  |
| *IFNG* |  |  |  |  | 265 (0.56) | 203 (0.53) | 62 (0.65) |  |  |  |  |
| rs3138557 |  |  |  |  | 182 (0.38) | 153 (0.40) | 29 (0.31) |  |  |  |  |
| (CA)_15_ |  |  |  |  | 30 (0.06) | 26 (0.07) | 4 (0.04) |  | 0.66 (0.44-0.98) | **0.037** |  |
|  |  |  |  |  |  |  |  |  |  |  |  |
| *IFNG* |  |  |  |  | 163 (0.34) | 136 (0.36) | 27 (0.28) |  |  |  |  |
| rs3138557 |  |  |  |  | 229 (0.48) | 181 (0.47) | 48 (0.51) |  |  |  |  |
| (CA)_16_ |  |  |  |  | 85 (0.18) | 65 (0.17) | 20 (0.21) |  | 1.25 (0.91-1.72) | 0.16 |  |
|  |  |  |  |  |  |  |  |  |  |  |  |
| *IFNG* |  |  |  |  | 415 (0.87) | 334 (0.87) | 81 (0.85) |  |  |  |  |
| rs3138557 |  |  |  |  | 61 (0.12) | 47 (0.12) | 14 (0.15) |  |  |  |  |
| (CA)_17_ |  |  |  |  | 1 (0.01) | 1 (0.01) | 0 |  | 1.17 (0.62-2.19) | 0.63 |  |
|  |  |  |  |  |  |  |  |  |  |  |  |
| *IFNG* |  |  |  |  | 414 (0.87) | 336 (0.88) | 78 (0.82) |  |  |  |  |
| rs3138557 |  |  |  |  | 60 (0.12) | 44 (0.11) | 16 (0.17) |  |  |  |  |
| (CA)_18_ |  |  |  |  | 3 (0.01) | 2 (0.01) | 1 (0.01) |  | 1.55 (0.88-2.71) | 0.13 |  |
|  |  |  |  |  |  |  |  |  |  |  |  |
| *IFNG* |  |  |  |  | 425 (0.89) | 341 (0.89) | 84 (0.88) |  |  |  |  |
| rs3138557 |  |  |  |  | 49 (0.10) | 39 (0.10) | 10 (0.11) |  |  |  |  |
| (CA)_19_ |  |  |  |  | 3 (0.01) | 2 (0.01) | 1 (0.01) |  | 1.12 (0.59-2.14) | 0.72 |  |
|  |  |  |  |  |  |  |  |  |  |  |  |
| *IFNG* |  |  |  |  | 442 (0.92) | 353 (0.92) | 89 (0.94) |  |  |  |  |
| rs3138557 |  |  |  |  | 33 (0.07) | 27 (0.07) | 6 (0.06) |  |  |  |  |
| (CA)_20_ |  |  |  |  | 2 (0.01) | 2 (0.01) | 0 |  | 0.78 (0.33-1.86) | 0.58 |  |
|  |  |  |  |  |  |  |  |  |  |  |  |
| *IFNG* |  |  |  |  | 237 (0.50) | 180 (0.47) | 57 (0.60) |  |  |  |  |
| rs3138557 |  |  |  |  | 189 (0.40) | 155 (0.41) | 34 (0.36) |  |  |  |  |
| (CA)_14/15_ |  |  |  |  | 51 (0.10) | 47 (0.12) | 4 (0.04) |  | 0.60 (0.42-0.87) | **0.007** |  |
|  |  |  |  |  |  |  |  |  |  |  |  |

**S2 Table. Association of the IFNG VNTR *rs3138557* with BU susceptibility.** OR stands for odds ratio, CI for confidence interval, All for total number of included individuals comprising Buruli ulcer (BU) patients and controls. Bold text indicates a difference with P<0.05.
